# Wirksamkeit digitaler Trainingstherapie bei patellofemoralen Schmerzen

**DOI:** 10.1007/s00132-025-04704-w

**Published:** 2025-08-08

**Authors:** Maleen Hinz, Daniel Wagner, Theresa Kölle, Tobias A. Mayer-Roth, Christian Maiwald

**Affiliations:** 1 Institut für Angewandte Bewegungswissenschaften, Professur Forschungsmethoden und Analyseverfahren in der Biomechanik, Technische Universität Chemnitz, Thüringer Weg 11, 09126 Chemnitz, Deutschland; 2 Hessingpark Clinic GmbH, Augsburg, Deutschland 86199; 3 Mawendo GmbH, Valley/Oberlaindern, Deutschland 83626

**Keywords:** Digitale Gesundheitstechnologie, Patientenberichtetes Ergebnismaß (KOOS), Patella, Physiotherapie, Selbstmanagement, Digital health technology, Patient reported outcome (KOOS), Patella, Physiotherapy, Self-management

## Abstract

**Hintergrund:**

Pathologien der Patella stellen ein häufiges Beschwerdebild im Bereich muskuloskelettaler Erkrankungen dar. Digitale Gesundheitsanwendungen (DiGA), wie Mawendo, bieten eine neue alternative Therapieform zur Physiotherapie.

**Ziel der Arbeit:**

In dieser Arbeit wurde die Wirksamkeit der DiGA Mawendo im Vergleich zur klassischen Physiotherapie anhand der KOOS-Subskalen bei patellofemoralen Beschwerden untersucht.

**Methoden:**

253 Teilnehmende wurden randomisiert einer DiGA- oder Physiotherapiegruppe über 12 Wochen zugewiesen. Primäre Endpunkte waren die KOOS-Subskalen, analysiert wurden diese deskriptiv und mittels ANCOVA.

**Ergebnisse:**

Die DiGA Mawendo erzielte in allen Subskalen signifikant bessere Ergebnisse mit Überschreitung der minimalen klinisch relevanten Differenz (MCID) für jede Subskala.

**Diskussion:**

Die DiGA zeigte einen klinisch relevanten, multidimensionalen Therapieeffekt über alle Subskalen. Ihre flexible Anwendung und hohe Effektivität sprechen für den (ergänzenden) Einsatz in der Regelversorgung.

**Graphic abstract:**

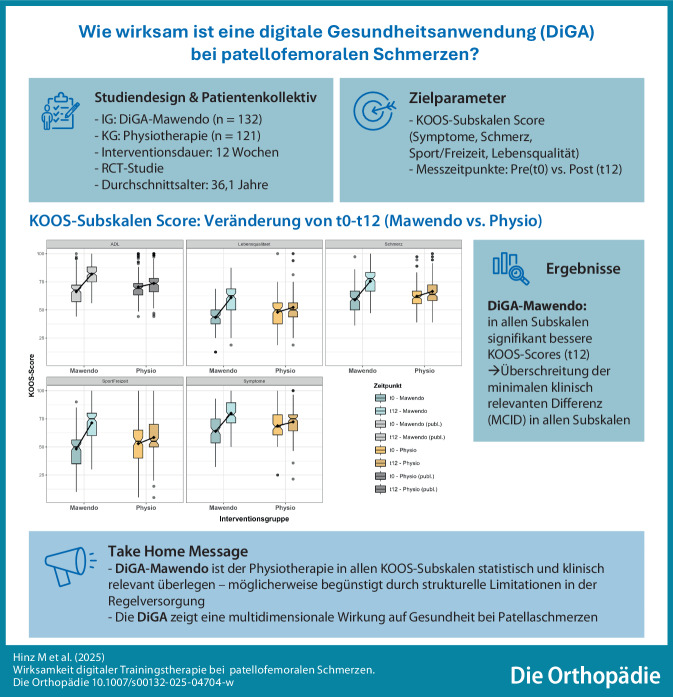

## Abstract (en)

**Background:**

Patellar pathologies are a common manifestation of musculoskeletal disorders. Digital health applications (DiGA), such as Mawendo, offer an alternative therapeutic approach to physiotherapy.

**Aim of the study:**

This study aimed to evaluate the effectiveness of the DiGA-Mawendo compared to conventional physiotherapy in patients with patellofemoral pain, based on the KOOS subscales.

**Methods:**

A total of 253 participants were randomly assigned to either a DiGA or physiotherapy group over a period of 12 weeks. The primary endpoints were the KOOS subscales, which were analyzed descriptively and using ANCOVA models.

**Results:**

DiGA-Mawendo showed significantly better outcomes in all subscales, exceeding the minimum clinically important difference (MCID) in each case.

**Discussion:**

DiGA demonstrated a clinically relevant, multidimensional therapeutic effect across all subscales. Its flexible use and high effectiveness support its complementary role in healthcare delivery.

Patellofemorale Beschwerden zählen zu den häufigsten Ursachen für Knieschmerzen. Digitale Gesundheitsanwendungen wie Mawendo bieten neue Ansätze in der konservativen Therapie. Diese Studie vergleicht Mawendo mit klassischer Physiotherapie anhand der KOOS-Subskalen Schmerz, Symptome, Aktivitäten des täglichen Lebens (ADL), Sport/Freizeit und Lebensqualität – mit signifikant besseren Ergebnissen für die digitale Anwendung.

## Einleitung

Muskuloskelettale Erkrankungen stellen in Deutschland eine der führenden somatischen Gesundheitsproblematiken dar. Sie verursachen erhebliche volkswirtschaftliche Kosten und können die Lebensqualität der Betroffenen stark beeinträchtigen [14]. Gelenkbeschwerden, insbesondere Knieschmerzen, treten häufig auf, wobei vor allem der vordere Kniebereich rund um die Patella betroffen ist [9]. Studien berichten über Prävalenzraten von patellofemoralen Beschwerden zwischen 15 und 45 % [5, 27, 28]. Pathologien der Patella sind unter dem Diagnoseschlüssel M22 (ICD-10-GM M22) gelistet [6] und werden unter dem klinischen Begriff des vorderen Knieschmerzes zusammengefasst. Dieses Beschwerdebild resultiert in der Regel aus einem multifaktoriellen Geschehen, das strukturelle, funktionelle und biomechanische Faktoren umfasst. Zu den relevanten Einflussfaktoren zählen unter anderem muskuläre Dysbalancen, Überlastung, anatomische Normvarianten wie Trochleadysplasie oder Beinachsenfehlstellungen [11, 13]. Eine zeitnahe und wirksame Therapie ist essenziell, um die Gelenkfunktion langfristig zu erhalten und Schmerzen zu reduzieren. In der Mehrheit der Studien zu M22-Diagnosen werden primär konservative Therapiekonzepte verfolgt, wobei physiotherapeutische und bewegungstherapeutische Interventionen im Vordergrund stehen. Diese beinhalten insbesondere trainingstherapeutische Maßnahmen zur Verbesserung der muskulären Funktion [1, 17, 18, 25]. Mit Inkrafttreten des Digitalen-Versorgungs-Gesetzes (DVG) im Jahr 2019 wurde die rechtliche Grundlage für die Integration digitaler Gesundheitsanwendungen (DiGA) in die Regelversorgung der gesetzlichen Krankenversicherung geschaffen. DiGA sind „digitale Helfer“, deren medizinischer Zweck im Wesentlichen auf digitalen Technologien beruht. Eine DiGA unterstützt die Erkennung, Überwachung, Behandlung oder Linderung von Krankheiten oder Verletzungen. Sie müssen die Anforderungen an ein digitales Medizinprodukt erfüllen. Anforderungen bezüglich Sicherheit, Funktionstauglichkeit, Qualität, Datenschutz, Informationssicherheit und positive Versorgungseffekte werden vom Bundesinstitut für Arzneimittel und Medizinprodukte (BfArM) geprüft. Seitdem sind mehrere DiGA in das Verzeichnis des BfArM aufgenommen worden [4, 16]. Aktuell sind drei DiGA zur Behandlung von Knieschmerzen gelistet, darunter die in der publizierten Studie von Mayer et al. (2025) genutzte browserbasierte Anwendung Mawendo [22]. DiGA bieten insbesondere im orthopädischen und unfallchirurgischen Kontext Vorteile, da sie eine orts- und zeitunabhängige Therapie ermöglichen und damit Versorgungsengpässen sowie Fachpersonalmangel entgegenwirken. Darüber hinaus unterstützen sie das Selbstmanagement der Patienten [7, 30]. Zur Evaluierung der Effektivität therapeutischer Interventionen werden in der Forschung standardisierte Erhebungsinstrumente eingesetzt. Bei Kniegelenkspathologien hat sich der Knee Injury and Osteoarthritis Outcome Score (KOOS) nach Roos et al. (1998) als valides Erhebungsinstrument etabliert [24]. Der KOOS besteht aus fünf Subskalen (Symptome, Schmerz, Aktivitäten des täglichen Lebens (ADL), Sport/Freizeit, Lebensqualität). In der prospektiven, randomisiert-kontrollierten Interventionsstudie von Mayer et al. (2025) wurde der Effekt der DiGA Mawendo im Vergleich zur klassischen Physiotherapie anhand des KOOS-ADL und der Visuellen Analogskala (VAS) untersucht [22]. Die hier vorliegende Arbeit baut auf dieser Studie auf und verfolgt das Ziel, eine vertiefte Auswertung der übrigen vier KOOS-Subskalen vorzunehmen, um den Therapieeffekt der digitalen Anwendung im Vergleich zur Physiotherapie differenzierter zu bewerten. Der in beiden Analysen verwendete KOOS basiert auf der deutschen Übersetzung von Kessler et al. (2003) [20].

## Methodik

Die Studie von Mayer et al. (2025) wurde am Institut für Angewandte Bewegungswissenschaften der Technischen Universität Chemnitz durchgeführt. Die Rekrutierung erfolgte zwischen 2021 und 2023 über orthopädische Facharztpraxen. Insgesamt wurden 253 Probanden im Alter ab 12 Jahren nach Prüfung der Ein- und Ausschlusskriterien randomisiert einer von zwei Gruppen zugewiesen: der Interventionsgruppe (DiGA Mawendo: *n* = 132) oder der Kontrollgruppe (Physiotherapie: *n* = 121). Teilnahmeberechtigt waren Personen ab 12 Jahren; bei Minderjährigen unter 18 Jahren war eine schriftliche Einverständniserklärung der Eltern erforderlich. Ausschlusskriterien umfassten relevante Vorerkrankungen (z. B. Operationen in den letzten 6 Monaten, akute oder komplexe Herz-Kreislauf- bzw. Atemwegserkrankungen, Tumoren, Infektionen/Fieber, muskuloskelettale Diagnosen außerhalb der M22-Kodierung, psychische Erkrankungen, Schwangerschaft, stark eingeschränktes Sehvermögen sowie erhöhte Blutungsneigung). Ebenso ausgeschlossen wurden Personen mit einem sehr geringen (VNRS ≤ 2) oder sehr starken (VNRS ≥ 8) Schmerzempfinden auf der Verbal Numerical Rating Scale (VNRS). Die Interventionsdauer betrug 12 Wochen.

**Abb. 1:**
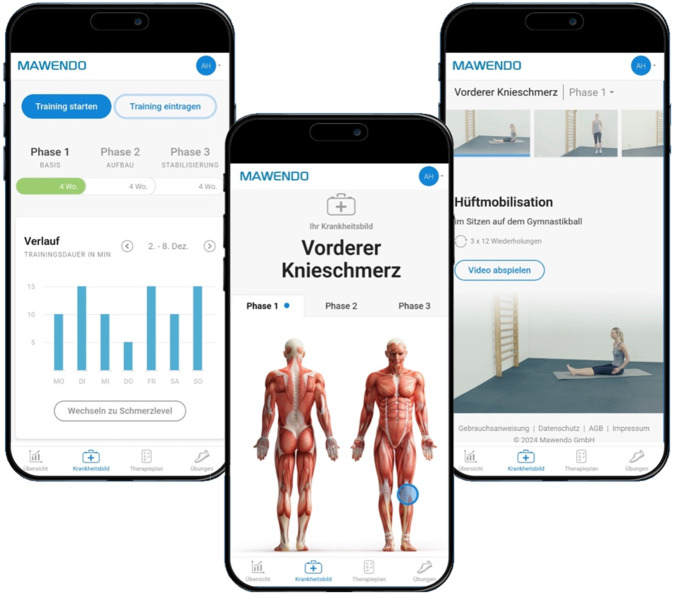
Screenshot der Benutzeroberfläche der DiGA Mawendo für die Indikation M22 (© Mawendo GmbH, mit freundlicher Genehmigung)

Die Probanden der Interventionsgruppe verwendeten die DiGA Mawendo (Mawendo GmbH, Valley/Oberlaindern, Deutschland), eine browserbasierte Anwendung, die aus digitalisierten Trainingsvideos besteht und durch visuelle sowie auditive Anleitungen ein strukturiertes Eigentraining ermöglicht (Abb. 1).

Die Anwendung ist in 3 progressiv aufgebaute Behandlungsphasen (Basis‑, Aufbau- und Stabilisierungsphase) unterteilt, die jeweils spezifische Übungen zur Mobilisation, Koordination, Massage, Dehnung und Kräftigung enthalten. Ein Training von 2–3 Einheiten pro Woche mit einer Dauer von 20–40 min wird empfohlen. Neben der Trainingsanleitung bietet die Anwendung auch Gesundheitsinformationen zur Erkrankung und eine Verlaufskontrolle. Die Trainingsinhalte orientieren sich an den Leitlinien der Arbeitsgemeinschaft der Wissenschaftlichen Medizinischen Fachgesellschaften (AWMF). Diese und weitere Studien zur Entwicklung der Trainingspläne sind auf der Website des Anbieters einsehbar [21]. Die Kontrollgruppe erhielt eine physiotherapeutische Standardversorgung gemäß Heilmittelkatalog [15]. Die physiotherapeutische Behandlung in der Kontrollgruppe erfolgte in Anlehnung an die aktuelle GKV-Versorgungspraxis mit maximal 12 Einheiten über 12 Wochen, entsprechend der im Heilmittelkatalog vorgesehenen Verordnungsmenge. Die konkrete Frequenz (z. B. 1–2 Behandlungen pro Woche) sowie die Inhalte wurden individuell mit dem behandelnden Therapeuten festgelegt. Eine strukturierte Heimübungskomponente wurde nicht standardisiert vorgegeben und nicht systematisch erfasst, konnte aber je nach Praxis Bestandteil der Therapieplanung sein.

Als primäres Outcome-Maß diente der KOOS, das mittels einer 5‑stufigen Likert-Skala kniespezifische Beschwerden erfasst und in einen Score von 0–100 transformiert wird (100 = keine Beschwerden). Der KOOS besteht aus 5 Subskalen, die den Gesundheitszustand der Betroffenen mehrdimensional abbilden. Die Subskala *Schmerz* erfasst die Intensität von Knieschmerzen in Ruhe, bei Belastung und alltäglichen Aktivitäten. Die Subskala *Symptome* bewertet Gelenksymptome wie Steifigkeit, Schwellung und Bewegungseinschränkungen. Mit der Subskala *ADL* werden funktionelle Einschränkungen im Alltag – etwa beim Gehen, Treppensteigen oder Aufstehen – erfasst. Auf der Subskala *Sport und Freizeit* werden Fähigkeiten zur Ausführung körperlicher Aktivitäten, wie Sport oder Hobbys mit Kniebelastung erfasst. Die Subskala *Lebensqualität* bildet die subjektive Wahrnehmung der Einschränkungen durch die Knieprobleme ab [20, 24]. Die Erhebung erfolgte vor Therapiebeginn (t0) und 12 Wochen nach Beginn (t12) der Intervention. Zur statistischen Auswertung wurden neben einer deskriptiven Analyse der Mittelwerte auch Mittelwertsdifferenzen berechnet und die relativen Effekte zur Einschätzung der klinischen Relevanz ermittelt. Darüber hinaus kamen für jede KOOS-Subskala lineare Modelle (ANCOVA) zur Anwendung, um Gruppenunterschiede zwischen DiGA- und Physiotherapiegruppe zu analysieren. Aufgrund der 4fachen einseitigen Testung wurde das Signifikanzniveau auf α = 0,00625 angepasst. Die Mittelwertsunterschiede zwischen t0 und t12 wurden zudem im Hinblick auf den minimalen klinisch relevanten Unterschied (MCID) interpretiert [19].

## Ergebnisse

In allen untersuchten Subskalen des KOOS zeigte sich ein statistisch signifikanter Therapieeffekt zugunsten der DiGA Mawendo im Vergleich zur konventionellen Physiotherapie. In der DiGA-Gruppe verbesserten sich die Mittelwerte über den 12-wöchigen Zeitraum hinweg. So stieg der Score in der Subskala Symptome von 63,9 auf 80 Punkte, in Schmerz von 59 auf 75,6 Punkte, in Sport/Freizeit von 48,3 auf 71,4 Punkte und in Lebensqualität von 43,4 auf 61,1 Punkte (Differenzen von 15,6 bis 23,2 Punkten). Die Kontrollgruppe mit konventioneller Physiotherapie wies dagegen geringe Verbesserungen auf – mit Differenzen von 4–6 Punkten pro Subskala (Tab. 1). Im Mittel konnte mit Mawendo ein etwa 4fach größerer Therapieeffekt erzielt werden als mit der physiotherapeutischen Standardbehandlung. Der stärkste deskriptive Effekt zeigte sich in der Subskala Lebensqualität, der schwächste in der Subskala Schmerz. Auch die inferenzstatistische Auswertung bestätigte die Überlegenheit der DiGA: Der adjustierte Gruppenunterschied in der ANCOVA war in allen Subskalen statistisch signifikant (Symptome: 10,7 P.; Schmerz: 10,6 P.; Sport/Freizeit: 15,1 P.; Lebensqualität: 11,1 P.; *p* < 0,0001).

**Tab. 1:** Deskriptive Ergebnisse der KOOS-Subskalen

Subskala	Intervention	Prä (t0)	Post (t12)	MW-Differenz	Relativer Effekt
Symptome	DiGA	63,9	80	16,1	4,3
	Physio	68,6	72,4	3,8	
Schmerz	DiGA	59	75,6	16,6	3,6
	Physio	62	66,6	4,6	
ADL^2^	DiGA	66,2	81,9	15,6	4,6
	Physio	70,3	73,7	3,4	
Sport/Freizeit	DiGA	48,3	71,4	23,2	3,9
	Physio	52,8	58,7	5,9	
Lebensqualität	DiGA	43,4	61,1	17,7	4,3
	Physio	48,1	52,2	4,1	

^1^ Dargestellt sind Mittelwerte, Mittelwertsdifferenzen (MW-Differenz) und der relative Effekt der KOOS(Knee Injury and Osteoarthritis Outcome Score)-Subskalen vor (t0) und nach (t12) der Intervention für jede Interventionsgruppe

^2^ Die Auswertung der Subskala ADL (Activities of Daily Living) war nicht Bestandteil dieser Arbeit, sondern findet sich in der Arbeit von Mayer et al. (2025) wieder

*DiGA* digitale Gesundheitsanwendung

**Abb. 2:**
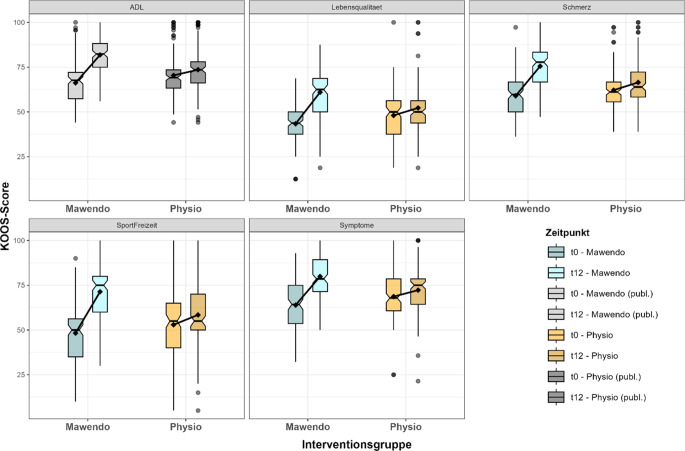
Veränderung der KOOS(Knee Injury and Osteoarthritis Outcome Score)-Subskalen vor und nach der Intervention in den Gruppen Mawendo und Physiotherapie. Dargestellt sind Boxplots mit dem Median (*schwarzer Strich* in der Box), dem 1. Quartil (*untere* Boxgrenze) und dem 3. Quartil (*obere* Boxgrenze). Die Whisker stellen den Wertebereich bis zum 1,5fachen des Interquartilsabstands dar. Ausreißer sind als *Punkte* markiert. *Schwarze Punkte* zeigen den Mittelwert, *schwarze Linien* visualisieren den Gruppenverlauf von vor (t0) zu nach der Intervention (t12). Die Subskala ADL (Activities of Daily Living) ist zur Übersicht mit in die graphische Darstellung aufgenommen, wurde aber in der Studie von Mayer et al. (2025) [22] bereits ausgewertet

Die Abb. 2 zeigt die KOOS-Scores in den einzelnen Subskalen zu den Messzeitpunkten t0 (vor der Intervention) und t12 (nach der Intervention), getrennt nach Interventionsgruppe. Die jeweiligen Daten werden mittels Boxplots dargestellt, wobei die schwarzen Punkte den Mittelwert der Scores markieren. Die Entwicklung über die Zeit wird zusätzlich durch eine verbindende Linie zwischen den Mittelwerten visualisiert, um die Richtung und das Ausmaß der Veränderung innerhalb der Gruppen anschaulich darzustellen.

## Diskussion

In allen KOOS-Subskalen zeigte sich ein statistisch signifikanter Therapieeffekt zugunsten der DiGA-Gruppe im Vergleich zur Kontrollgruppe (Regelversorgung GKV-Physiotherapie). Besonders ausgeprägt war die Verbesserung in der Subskala Lebensqualität, was auf einen umfassenden therapeutischen Nutzen der digitalen Intervention hinweist, der über rein funktionelle Aspekte hinausgeht. Die Ergebnisse legen nahe, dass Patienten nicht nur eine Verringerung von Symptomen und Schmerzen erfahren, sondern auch eine spürbare Steigerung ihrer subjektiv empfundenen Lebensqualität. Dies ist insbesondere bei länger andauernden Beschwerden von Bedeutung, die potenziell chronifizieren und mit psychischer Belastung einhergehen können. Auch in der Subskala Sport/Freizeit wurden signifikante Verbesserungen festgestellt, die auf eine gesteigerte Teilhabe an alltäglichen, sportlichen und sozialen Aktivitäten hinweisen. Dies könnte langfristig zur Stabilisierung des Aktivitätsniveaus beitragen und sekundäre Folgeprobleme wie Inaktivität, Gewichtszunahme oder sozialen Rückzug reduzieren [29]. Die Subskalen Schmerz und Symptome zeigten ebenfalls signifikante Effekte. Die Schmerzreduktion ist eines der vorrangigen Ziele in der konservativen Therapie patellofemoraler Beschwerden [10]. Zusätzlich deutet eine Verbesserung von Symptomen wie Schwellung oder Steifigkeit auf eine mehrdimensionale Wirkung der DiGA hin. Für die Interpretation der Mittelwertsunterschiede zwischen den Messzeitpunkten ist eine Einordnung im Hinblick auf die klinische Relevanz unerlässlich. Für die vorliegende Analyse wurden die von Nishimoto et al. (2024) publizierten MCID-Werte berücksichtigt [23], welche je nach Subskala wie folgt ausfallen: Symptome: 9 P, Schmerz: 13 P, Sport und Freizeit: 9 P, Lebensqualität: 16 P. In allen Subskalen überschritten die beobachteten Veränderungen (t0 zu t12) der vorliegenden Arbeit diese Schwellenwerte deutlich. Diese Ergebnisse stimmen mit den Ergebnissen anderer Studien zur Wirksamkeit digitaler Gesundheitsanwendungen überein. Correia et al. (2019) zeigten nach Knietotalendoprothesen signifikante Vorteile der digitalen Trainingsintervention in allen KOOS-Subskalen gegenüber konventioneller Rehabilitation. Dieser Effekt zeigte sich auch deutlich in den berechneten Differenzen der Änderungen des Medians zwischen den Gruppen (Lebensqualität: 37 P; Schmerz: 23,5 P; Symptomen: 21,5 P; Sport/Freizeit: 15 Punkte) [8]. Dieter et al. (2024) fanden in einer Pilotstudie zu Kniearthrose ebenfalls signifikante Verbesserungen zugunsten der DiGA-Gruppe. Die berechneten Differenzen der Mittelwertsveränderungen zwischen DiGA- und Kontrollgruppe lagen bei 18,4 P (Schmerz), 14,5 P (Symptome), 10,5 P (Sport/Freizeit) und 13 P (Lebensqualität) [12]. Ähnliche Ergebnisse berichten Schmidt et al. (2025) in einer randomisierten kontrollierten Studie zur Wirksamkeit der „Orthopy“-App in der Rehabilitation nach einer Kreuzbandruptur. Während sich die Subskalen Schmerz und Symptome zu mehreren Zeitpunkten signifikant verbesserten, zeigten die Subskalen Sport/Freizeit und Lebensqualität in der postoperativen Phase keine signifikanten Veränderungen. Dies könnte auf den kurzen Beobachtungszeitraum zurückzuführen sein, da funktionelle Teilhabe und psychosoziale Aspekte typischerweise erst im späteren Verlauf der Rehabilitation nach einer Kreuzbandoperation signifikante Veränderungen zeigen [26].

Diese Studien unterstreichen die Effektivität von digitalen Therapieprogrammen und deuten auf einen in der Versorgungspraxis relevanten Therapieeffekt hin, der sich nicht nur in einer Reduktion von Schmerz und Symptomen zeigt, sondern multidimensional positiv auf die Gesundheit der Betroffenen wirkt. Vor dem Hintergrund dieser Ergebnisse erscheint ein Vergleich mit der klassischen Physiotherapie, deren Maßnahmen in den Heilmittelrichtlinien § 92 Abs. 1 Satz 2 Nr. 6 SGB V definiert sind, angebracht. Strukturelle Einschränkungen wie kurze Behandlungszeiten, begrenzte Behandlungsfrequenzen und standardisierte Verordnungsmengen könnten die Wirksamkeit physiotherapeutischer Maßnahmen einschränken [2, 3]. Digitale Trainingsinterventionen bieten demgegenüber Vorteile wie ein höheres Trainingsvolumen, orts- und zeitunabhängige Nutzung, visuelle Anleitung und die Förderung von Selbstmanagement, wodurch Versorgungslücken reduziert und Therapieeffekte verstärkt werden können [3, 7, 30].

Eine Einschränkung der vorliegenden Studie besteht in der fehlenden Langzeitbeobachtung, da über den 12-Wochen-Interventionszeitraum hinaus keine Follow-up-Erhebungen durchgeführt wurden. Zudem beruhen die Ergebnisse ausschließlich auf patientenberichteten Outcome-Maßen (PROM), wodurch potenzielle subjektive Verzerrungen nicht ausgeschlossen werden können. Eine Verblindung der Teilnehmenden war aufgrund des gewählten Studiendesigns nicht realisierbar. Durch die randomisierte Zuteilung zu den Interventionsgruppen konnten jedoch potenzielle systematische Verzerrungen zwischen den Gruppen kontrolliert und die Auswirkungen dieser methodischen Limitationen weitgehend minimiert werden. Darüber hinaus ist zu berücksichtigen, dass Trainingsfrequenz und -intensität zwischen Interventions- und Kontrollgruppe nicht vollständig vergleichbar waren. Die Anzahl physiotherapeutischer Behandlungen in der Kontrollgruppe variierte abhängig von der ärztlichen Verordnung, der Praxisorganisation und dem Patientenverhalten. Eine systematische Erfassung etwaiger Heimübungen erfolgte nicht. Unterschiede in der Trainingsfrequenz oder -qualität könnten daher das Ergebnis zugunsten der Interventionsgruppe beeinflusst haben. Vor diesem Hintergrund kann nicht davon ausgegangen werden, dass ein digitales Trainingsprogramm eine qualifizierte physiotherapeutische Behandlung vollständig ersetzen kann.

## Fazit für die Praxis

- Die Digitale Gesundheitsanwendung (DiGA) Mawendo war der klassischen Physiotherapie in allen untersuchten KOOS(Knee Injury and Osteoarthritis Outcome Score)-Subskalen (Symptome, Schmerz, Activities of Daily Living, Sport/Freizeit, Lebensqualität) statistisch signifikant und klinisch relevant überlegen.
- Der größte Therapieeffekt zeigte sich in der Subskala Lebensqualität, der geringste – wenngleich signifikant und klinisch relevant – in der Subskala Schmerz.
- Die DiGA Mawendo ermöglicht ein strukturiertes Eigentraining mit höherem Trainingsvolumen und fördert das Selbstmanagement – zentrale Faktoren zur Verbesserung funktioneller und psychosozialer Gesundheitsaspekte.
- Begrenzte Therapiedauer, niedrige Behandlungsfrequenzen und standardisierte Verordnungsmengen könnten die Effektivität klassischer Physiotherapie einschränken.

## Data Availability

Die zugrundeliegenden Daten wurden im Rahmen einer klinischen Studie erhoben, die von den mitverantwortlichen Autoren durchgeführt wurde. Eine Weitergabe der Daten unterliegt ethischen und datenschutzrechtlichen Vorgaben sowie institutionellen Regelungen. Anfragen zur Datennutzung können ggf. an die korrespondierenden Autoren gerichtet werden.

## Abkürzungen

ADL: Aktivitäten des täglichen Lebens
AWMF: Arbeitsgemeinschaft der Wissenschaftlichen Medizinischen Fachgesellschaften
BfArM: Bundesinstitut für Arzneimittel und Medizinprodukte
DiGA: Digitale Gesundheitsanwendung
DVG: Digitalen-Versorgungs-Gesetzes
GKV: Gesetzliche Krankenversicherung
IG: Interventionsgruppe
KG: Kontrollgruppe
KOOS: Knee Injury and Osteoarthritis Outcome Score
MCID: Minimal Clinical Important Difference
PROM: patientenberichtete Outcome-Maße
RCT: Randomized Controlled Trial
VAS: Visuelle Analogskala
VNRS: Verbal Numerical Rating Scale
